# Physiological Mechanisms in Pregnancy and Their Relevance to the Clinical Management of Perinatal Mental Illness

**DOI:** 10.3390/jcm15124559

**Published:** 2026-06-12

**Authors:** Annemarie Unger, Nora Rosenberg, Alexandra Kautzky-Willer, Alexander Kautzky

**Affiliations:** 1 Clinical Division of Social Psychiatry, Department of Psychiatry and Psychotherapy, Medical University of Vienna, 1090 Vienna, Austria; annemarie.unger@meduniwien.ac.at (A.U.); nora.rosenberg@meduniwien.ac.at (N.R.); 2 Comprehensive Center for Clinical Neurosciences and Mental Health, Medical University of Vienna, 1090 Vienna, Austria; 3 Division of Endocrinology and Metabolism, Department of Internal Medicine III, Medical University of Vienna, 1090 Vienna, Austria; alexandra.kautzky-willer@meduniwien.ac.at; 4 Division of Insurance Medicine, Department of Clinical Neurosciences, Karolinska Institute, 171 77 Stockholm, Sweden

**Keywords:** perinatal psychiatry, postpartum, maternal mental health

## Abstract

Perinatal mental illness affects up to 20% of new mothers worldwide, yet despite a growing research interest over the past decade, the etiology is still not fully understood, and clinical treatment guidelines remain inconsistent across countries and services. In this review, recent findings on neurobiological processes and evolutionary mechanisms, as they occur during the menstrual cycle, pregnancy, birth, postpartum and breastfeeding, are discussed. The intention is to raise awareness of physiological changes in pregnancy that might be relevant to the differential diagnosis and clinical treatment of perinatal psychiatric disorders such as depression, anxiety, PTSD after childbirth, bipolar relapse, postpartum psychosis, obsessive-compulsive symptoms, substance-use disorders, and suicidality. Areas addressed include the activities of the immune system, thyroid gland, cortisol, sleep and individual sensitivity to ovarian hormone fluctuations. Evolutionary biological mechanisms intended to sustain pregnancy and to ensure the survival of the newborn are assumed to have potent effects on the maternal brain. These non-pathological adaptations could provide grounds for a better understanding of risk factors and the etiology of perinatal mental illness.

## 1. Introduction

Perinatal psychiatric illness is a global health problem affecting up to 20% of childbearing women [1]. Non-psychotic perinatal mental health conditions like depression and anxiety are common and affect about 10% of women in high-income countries and 20% in middle- and low-income countries [1]. Psychotic perinatal health conditions like schizophrenia, affective psychosis, and forms of bipolar disorders are less common and affect about 1–3 per 1000 births [2]. Untreated perinatal mental health conditions can increase morbidity and mortality rates for mothers and infants [3,4,5]. Affected women tend to have poor physical and psychological health, difficulties in inter-personal relationships, vulnerability to substance abuse and suicidality [5,6,7], with unintentional drug overdoses and suicide accounting for about one fifth of perinatal deaths in the United States [8].

Effects on offspring may start in utero, with infants of mothers with antenatal depression being more likely to be born pre-term and with a lower birth weight [9]. After birth, these infants are more vulnerable to poor developmental trajectories and internalizing and externalizing problems, in addition to a higher likelihood of future psychopathology [10,11]. Perinatal mental health symptoms thus require timely recognition, assessment and treatment, as outlined by the NICE and ACOG guidelines [12,13,14]. To ensure adequate treatment settings, it is important that perinatal mental health services are integrated within maternal and child health care settings and that psychiatric mother–baby units, which are considered the best clinical care model, are available to mothers needing inpatient treatment [1,15]. Though specialization in perinatal psychiatry has become more common, many non-specialized psychiatrists are reluctant to treat pregnant women and postpartum mental illness due to a lack of routine and knowledge. Often little is known about physiological responses and mechanisms in pregnant patients. To date, many reviews have focused on psychosocial risk factors for perinatal mental health conditions, while fewer integrate endocrine, immune, sleep, thyroid, and birth-related mechanisms. Furthermore, there is a clinical knowledge gap on practical frameworks for screening, risk stratification, pharmacological decisions, sleep protection, breastfeeding support, and mother–infant care. The present narrative review summarizes physiological mechanisms across reproductive stages and discusses their relevance for the prevention, screening, and clinical management of perinatal mental illness. Most research on perinatal mental health has historically focused on postpartum depression; accordingly, this review draws on many clinical examples related to this condition, while also including a broader range of non-psychotic and psychotic perinatal mental health disorders.

## 2. Methods

We conducted a narrative literature review on physiological mechanisms relevant to perinatal mental illness. PubMed, Scopus, and Google Scholar were searched from 2006 to 2026 using combinations of terms including pregnancy, postpartum, perinatal depression, postpartum psychosis, ovarian hormones, allopregnanolone, cortisol, thyroid, immune system, cytokines, sleep architecture, breastfeeding, oxytocin, prolactin, birth trauma, lactation and skin-to-skin contact. Specific mechanisms were selected based on their clinical relevance to perinatal mental illness. Only English-language articles were included. The focus was on postpartum psychiatric syndromes. Priority was given to systematic reviews, meta-analyses, randomized controlled trials, large cohort studies, and mechanistic studies in humans. Preprints were not included. Animal studies were included only when human evidence was limited and were explicitly labeled as preclinical evidence. Guidelines were included as references. Of over 150 articles included, only about 7 were published prior to the year 2000.

## 3. The Female Sex Steroids: Estrogen and Progesterone

### 3.1. Estrogen

Estrogens are neurosteroids synthesized from cholesterol in the ovaries and, to a much lesser degree, in a range of other organs, including the brain. They are produced by neurons and astrocytes, particularly in the hippocampus, cerebellum, hypothalamus, cortex, and amygdala [16]. Estrogens exert potent effects on mood and cognition by modulating serotonergic, glutamatergic, dopaminergic, cholinergic, noradrenergic, neuropeptide-Y, and opioidergic systems [17,18,19,20].

Effects occur via estrogen receptors as genomic mechanisms, as well as via membrane receptors as intracellular messenger signals, directly changing cellular function through rapid membrane effects [17,18,21,22]. Estrogens unfold antidepressant, neuroprotective and anti-inflammatory effects through several mechanisms. Antidepressant effects are promoted through serotonergic and dopaminergic pathways—serotonin production is increased, and the serotonin transporter (SERT) is modulated, lowering pain transmission [23]. Furthermore, dopamine transmission is enhanced, though direct effects on the dopamine receptor can be both stimulating and inhibitive [24]. Neuroprotective effects are mediated partly through upregulation of brain-derived neurotrophic factor (BDNF), which promotes neuroplasticity. Finally, estrogens modulate immune function by shifting cytokine ratios during pregnancy [25]. Clinical, animal and genetic studies suggest fluctuations in sex hormones, particularly estrogen, during the premenstrual, peripartum and perimenopausal phase, bring along vulnerability to depression [26]. Currently, there are no established biomarkers for the clinical identification of this “reproductive subtype” of depression; therefore, identification relies on careful clinical interviewing, which should consider the timing of depressive episodes and a personal or family history of depression associated with reproductive events [26]. This may have implications for pharmacological treatment since several RCTs have reported a quick and preferential response to antidepressant treatments with selective serotonin reuptake inhibitors (SSRIs) among premenopausal women, compared to postmenopausal women and men [27]. A recent meta-review of 14 randomized controlled trials (RCTs) on the administration of exogenous estrogens showed significant antidepressant effects among women with depression during the perimenopause or postpartum stages or with premenstrual dysphoric disorder [28]. Despite these promising results, clinical utility for perinatal psychiatry is currently limited by the small number of postpartum participants in these studies and the side effects of estrogen, such as increased risk for thrombosis in the first six weeks postpartum [29,30,31].

### 3.2. Progesterone

Progesterone is a cholesterol derivative, synthesized in the adrenal gland and corpus luteum of the ovary, effecting endometrial proliferation. In pregnancy, production also occurs in the placenta, upholding pregnancy by promoting cervical closure, myometrial relaxation and hindrance of labor. As a neurosteroid, synthesized by neurons and glial cells, it accelerates turnover of serotonin, reduces pain transmission, and has sedative and neuroprotective properties [32,33,34]. It also influences estrogen activity and, like estrogen, exerts its effects via genomic and non-genomic mechanisms [19,35]. Findings on absolute levels of progesterone and its most prominent metabolite, allopregnanolone, in relation to psychiatric symptoms such as depression and anxiety have been inconclusive, and several studies present fluctuations in progesterone and estrogen, rather than their absolute level, as the causal factor for psychiatric symptoms in sensitive individuals [36,37].

Exogenous progesterone, called progestin, is most commonly administered in the form of contraceptives, providing a natural opportunity to study the mental health effects of progesterone administration in large epidemiological studies. Although the effects of contraceptives on mood have been inconclusive, one nationwide cohort study including over 1 million women in Denmark associated use of oral contraceptives with subsequent use of antidepressants [38]. A recent Swedish longitudinal cohort study found that self-reported mood swings during contraceptive use predicted depression during pregnancy and doubled the risk of postpartum depression [39]. This has implications for clinical interviewing, as asking about previous mood effects of oral contraceptive therapies may help identify women who are at risk [39]. Derivatives of the progesterone metabolite allopregnanolone seem to have differential mental health effects from progestin and offer new treatment options in perinatal psychiatry. For postpartum depression, the FDA has recently approved two allopregnanolone analogues, the i.v. formulation Brexanolone (*Zulresso*) in 2019 and the oral drug Zuranolone (*Zurzuvae*) in 2023. Both drugs modulate gamma-aminobutyric acid (GABA), a receptor with anxiolytic, hypnotic/sedative, anesthetic and anticonvulsive effects [40,41,42,43,44]. A recent meta-analysis of RCTs on the safety and efficacy of these new “GABAkines” has confirmed positive effects on the core symptoms of postpartum depression compared with placebo [45].

## 4. Pre-Conception and Menstrual Cycle Vulnerability

The cyclical variability of hormone levels during the menstrual cycle has been proposed to influence the affective, cognitive and behavioral components of mental disorders to a varying degree, depending on susceptibility [46,47]. Increasing evidence points towards dynamic functional and morphologic changes in the brain and respective behavior in relation to the menstrual cycle and sex steroid levels [48,49]. Most women of childbearing age experience a better mood at the time of ovulation, when estrogen levels peak [50]. Ovulation has been identified as the optimal time for learning new motor skills [51]. Psychiatric symptom exacerbation most often occurs when estrogens and progesterone are low, as is the case during the premenstrual phase and menstruation [52]. In inpatient psychiatric settings, women will frequently be admitted in the premenstrual phase and be ready for dismissal soon after their period is over [53,54,55].

Cyclical effects in the sense of symptom aggravation during premenstrual and menstrual phases have been confirmed for schizophrenia [56,57], bipolar disorder [58,59], major depression [60,61], anxiety, PTSD [62], ADHD [63], alcohol abuse [64], suicide attempts and suicide [65,66,67,68]. In the DSM-V, the new diagnosis of premenstrual dysphoric syndrome has been defined since 2012, with a point prevalence of 1.6% among premenopausal women [69,70]. Based on the importance of the menstrual cycle for the development, presentation and treatment of psychiatric symptoms in women, obtaining information on cycle history should be included in psychiatric evaluations of premenopausal patients.

## 5. Pregnancy-Related Immune, Endocrine and Circadian Adaptations

### 5.1. The Immune System

Implantation of the egg occurs in a pro-inflammatory state with the aid of cytokines contained in seminal plasma fluid that influence endometrial cells. The maternal immune system receives signals from the trophoblast, the outer layer of the embryo. These signals can influence the development of immune cells, such as macrophages/monocytes, into trophoblast-supporting phenotypes. These complex mechanisms allow for a temporary transformation into an anti-inflammatory state in the second trimester to avoid rejection of the fetus [71,72]. In the third trimester, a pro-inflammatory state recurs. Based on evidence for inflammatory processes contributing to psychiatric illness, the shift to a pro-inflammatory state occurring just before birth has been postulated as a risk factor for the development of postpartum depression [73,74]. Non-perinatal mental disorders have previously been associated with immune dysregulation such as increases in pro-inflammatory cytokine levels (IL-1, IL-6, TNF-α, and chemokines) [25,75,76].

Along these lines, inflammatory conditions during pregnancy, such as pre-eclampsia, gestational diabetes, rheumatic diseases and urinary tract infections, are associated with higher rates of peripartum depression and anxiety [77,78,79,80,81]. Divergent patterns of antenatal immune responses and immune cell ratios were identified for women with perinatal anxiety compared to mentally healthy women [82]. Several studies have investigated cytokine signatures as molecular biomarkers for postpartum anxiety and depression; however, none have yet demonstrated clinically meaningful value in the identification, prediction or stratification of perinatal psychiatric illness [83].

Although the directionality of these links is not clear, evidence supports routine anxiety and depression screening for pregnant women with inflammatory conditions.

### 5.2. The Thyroid Gland

Thyroid hormones play an important role in adult brain functions such as cognition and mood [84] and also have a crucial function in the development of the fetal and neonate brain [85]. An association between altered thyroid function and impaired mental health has been well established [86].

In pregnancy, ß-HCG stimulates the production of thyroid-stimulating hormone (TSH), and the thyroid gland increases in size and upregulates blood flow and output to accommodate the needs of the pregnant body and the growing fetus. The amount of thyroid-binding globulin (TBG) in late pregnancy is influenced by the level of estrogen and heightened levels have been linked to postpartum depression [87]. Hashimoto’s disease is linked to mental symptoms and heightened risk of pregnancy complications such as postpartum hemorrhage. Hashimoto’s encephalopathy is a rare complication that has been found to be linked to postpartum psychosis [88,89,90,91]. One recent study found a likelihood of any thyroid disorder in postpartum psychosis compared to mentally healthy postpartum women to be 17% vs. 3% [91]. Usage of thyroid hormones as biomarkers/predictors of postpartum depression was attempted in several prior studies [83,92,93]. However, levels of TPO antibodies had limited predictive value for postpartum depression [87,94], suggesting that this mechanism is relevant for a subset rather than the whole clinical population. Clinical thyroid screening through blood samples is thus essential for women presenting with perinatal psychiatric symptoms, ensuring that thyroid dysfunctions are identified, and affected women receive endocrine consultations and treatment.

### 5.3. Cortisol and Pregnancy

Pregnancy is characterized by heightened cortisol levels, as by the end of the third trimester, the placenta promotes corticotropin-releasing hormone (CRH) to a four times higher level compared to non-pregnancy. This hypercortisolism is transient and is obligatory for fetal brain development. Significant differences in postpartum drops of cortisol levels have been detected in women with postpartum depression compared to healthy controls [95]. Increased maternal cortisol levels have been linked to an increased likelihood of psychiatric disorders in the offspring [96,97]. But this association has only been empirically verified in a small number of observational studies, so the underlying mechanisms of maternal stress on child outcomes need to be further explored [98]. A growing body of evidence supports the negative effects of untreated mental illness on maladaptive developmental programming within the maternal-fetal unit. The dyad is to be understood as a unit, particularly regarding the influence of maternal conditions such as cardiovascular risk and gestational diabetes on fetal growth. This applies to the function of stress hormones and the immune system, which may affect the neurodevelopmental and cardiovascular outcome of the newborn [99]. Excessive maternal stress that can be caused by untreated perinatal mental conditions may negatively impact the health of the offspring.

Studies attempting to use CRH levels in pregnancy as a biomarker/predictor for postpartum depression have been inconclusive [83,100,101,102,103], and measurements of salivary cortisol levels in pregnancy failed to identify biomarkers [104,105]. Therefore, HPA-axis measures currently remain research tools and do not serve as clinical biomarkers.

### 5.4. Sleep Architecture in Pregnancy and the Risk of Sleep Deprivation

At the beginning of pregnancy, rising levels of progesterone will cause sedation in most women. Rising estrogen levels diminish the amount of restful non-rapid eye movement (REM) sleep throughout pregnancy, with a peak toward the third trimester, so that more awakenings take place [106]. The increase in duration of REM sleep during pregnancy might serve to enhance wakefulness by modulating noradrenergic receptors [107,108]. Discontinued sleep may have the evolutionary benefit of preparing for heightened vigilance, enabling mothers to tend to the frequent small feedings of the newborn. Furthermore, wakefulness, through cortical arousal, could be a safety measure for hearing every movement and respiratory event of the baby.

However, excessive pregnancy-related sleep disturbance and a significant increase in the occurrence of parasomnias such as restless legs syndrome [109] have also been associated with pre-term labour and gestational diabetes among other complications [110]. Furthermore, the relationship between sleep and psychiatric disorders is bi-directional [111,112]. While sleep disturbances are commonly reported by patients with any psychiatric disorder, impaired sleep can also promote the development of affective disorders by stimulating norepinephrine, which activates pathways to neuroinflammation [113]. Both impaired sleep quantity and quality can contribute to affective disorders. While short and excessive sleep duration may be detrimental, particularly sleep under 6 h daily has been associated with major depression [114]. Regarding sleep architecture, shifts in the ratio of non-REM to REM sleep are also seen in non-pregnancy-related depression and have been identified as predictors thereof. As part of the diagnostic criteria for major depression in the DSM-V, the shift in sleep architecture is comparable to the one in pregnancy [115,116]. Some of the changes in sleep quality experienced during pregnancy are permanent and do not fully reverse [110]. Many antidepressants influence sleep architecture by suppressing REM sleep. In bipolar disorder, pregnancy is particularly challenging, as poor tolerance of sleep deprivation poses a risk for postpartum psychosis [117,118]. The link between postpartum psychosis and bipolar disorder has been substantiated. Approximately 50% of women affected by postpartum psychosis will develop episodes within the bipolar spectrum outside of pregnancy and have a high risk of relapse after subsequent pregnancies [119]. Autoimmune thyroiditis and infections should be ruled out as treatable factors [88,120]. Clinically, these findings underscore the need for proactive sleep protection in patients with bipolar disorder and individuals at risk of postpartum psychosis. Interventions for maintaining sleep stability, such as sleeping medication and social support with infant night care, may reduce the risk of relapse and improve overall outcomes.

## 6. Birth and Risk of Birth Trauma

Compared to other primates, human birth is of long duration and poses risks for both mother and neonate due to the significant disproportion between the large fetal head and the narrow pelvis evolved from bipedalism [121]. The initiation of parturition is carefully timed, as both pre-term and post-term birth are associated with medical complications and neonatal mortality. Oxytocin is the main initiator of labor [122]. Physiological birth is a state of altered consciousness with temporary hypofrontality and a decrease in communication as labor progresses [123].

Recent years have been marked by a continuous increase in medical interventions in childbirth [124]. These frequently involve epidural anesthesia for pain management, interfering with the natural course of contractions, leading to applications of synthetic oxytocin. Exposure to synthetic oxytocin often results in hyper-stimulation of the uterus, instrumentally assisted birth, and has been identified as a risk factor for postpartum depression and PTSD [125,126]. Emergency cesarian delivery may become necessary, which, together with planned cesarian delivery, has put C-section rates on a continuous rise, up to 32% in countries such as Austria and the US [127,128].

Studies have shown that the mode of delivery has a profound effect on the postpartum mental health of mothers [129,130]. In an observational longitudinal study, a group of 685 mothers was monitored for psychopathology during the first 3 months postpartum. Women with C-sections and instrumentally assisted vaginal births developed higher rates of psychiatric symptoms than women who had natural vaginal births. This became particularly evident for those who had unplanned cesarians [130].

Perceived birth trauma affects 20% to 45% of women worldwide and has become so prominent that it has received its own terminology, post-traumatic stress disorder following childbirth (PTSD-FC) [131]. PTSD-FC has been identified as a risk factor for postpartum depression [132], and rates increase after birth complications, emergency delivery and prior trauma. The postpartum phase is a risk period for re-activation of prior PTSD, which can hinder regular mother-to-child bonding [133]. Studies show that prior PTSD has a worse effect on bonding than PTSD-FC. Clinically, these findings underscore the importance of trauma-informed maternity care and awareness of childbirth-related PTSD to improve maternal and infant mental health outcomes.

## 7. Postpartum

In pregnancy, 1000-fold rising levels of estrogens lead to changes in dopamine transmission. Rodent experiments have demonstrated that the availability of abundant estrogen changes dopamine receptor (D2) and dopamine transporter (DAT) density via rapid non-genomic membrane mechanisms [134]. Heightened estrogen and progesterone levels suddenly drop to pre-pregnancy amounts within five days postpartum. In rodent models, this “estrogen-withdrawal” state results in increased alertness and anxiety. From an evolutionary standpoint, one might speculate that the condition may have had the purpose of promoting the safety of the baby by lowering risk-taking behavior [135,136]. It may, however, also contribute to sleep disturbance for vulnerable individuals. Stress of childcare, coupled with sleep deprivation, heightens the risk for perinatal mental disorders. Along these lines, animal models also point towards behavioral changes induced by lactation and hormones such as oxytocin and prolactin that promote aggressive behavior in females [137]. While both in animals and humans, aggression is far more frequent in males than in females, increased maternal aggression likely serves similar evolutionary goals of protecting offspring and competing for resources such as food [138]. Implications for peripartum psychiatry are still insufficiently understood, but evidence from basic research in animal models is suggestive of potential glutamatergic targets in areas such as the ventrolateral hypothalamus, which is particularly involved in female or maternal aggression [139,140].

## 8. Breastfeeding and Bonding

Oxytocin and prolactin are inhibitory for stress responses, and both are essential in lactogenesis and milk ejection. Furthermore, they serve to protect the lactating mother from overreacting to stressors. In fact, the presence of a high level of prolactin increases maternal patience and reduces stress as she is less reactive to external demands [141,142]. Production of prolactin is increased by estrogen and TRH and decreased by dopamine via signaling to the pituitary gland. Despite well-established health benefits and the WHO recommendation for exclusive breastfeeding during the first six months [143], many women affected by severe perinatal psychiatric illness do not breastfeed or exhibit shorter duration or non-exclusive breastfeeding, so the potential stress protection of high prolactin levels is not present [144]. Besides social and cultural factors, such as hospital practices and lifestyle factors, depression itself is a key obstacle to breastfeeding. Several recent studies have proposed a bidirectional relationship between depressive symptoms and breastfeeding behavior [145,146,147,148,149,150,151]. External or intrinsic pressure to breastfeed can also contribute to anxiety and depression among mothers who decided against breastfeeding [152]. In a clinical context, breastfeeding recommendations should thus be individualized to the situation and preferences of women [153]. Women with perinatal psychiatric conditions should have access to lactation counseling and, when possible, be supported in their decision to breastfeed or not to breastfeed.

By reducing stress and promoting well-being, the release of oxytocin also plays an important role in mother-to-child bonding [154]. Some studies have found oxytocin levels in the third trimester to be predictive of postpartum depression; however, this only applies to a short time frame of the last two weeks of pregnancy to within the first two weeks postpartum. A sharp decline in oxytocin levels from week 38 to 2 days after delivery was found for women who developed postpartum depression, whilst healthy ones had a continuous increase in oxytocin levels [56,154,155,156]. RCTs investigating the effects of exogenous oxytocin administration on maternal mental health have so far produced inconsistent findings regarding depressive symptoms [157]. Oxytocin can also be stimulated naturally through skin-to-skin contact between mothers and infants [158], with strong clinical evidence for infant health benefits such as better thermoregulation, blood glucose levels, and cardiorespiratory stability and improved suckling. Benefits for maternal health include earlier expulsion of the placenta and reduced bleeding [159]. Furthermore, RCTs on maternal mental health outcomes suggest skin-to-skin contact may reduce maternal stress and anxiety during the early postpartum period, with less consistent evidence for a reduction in depressive symptoms [160,161]. The effects of skin-to-skin contact have not been specifically examined in RCTs in clinical samples with perinatal mental illness, and this is unlikely to happen since the authors of the most recent Cochrane review have advised against future trials withholding skin-to-skin contact in the control arm [159]. Based on evidence from non-clinical samples, women experiencing perinatal mental illness should be encouraged and supported to practice skin-to-skin care when desired, provided there are no clinical contraindications.

## 9. Discussion and Clinical Translation

Despite the fact that the past three decades have yielded a substantial number of new publications on perinatal mental illness, the complete understanding of its etiology is still in the works [162]. This review concludes that physiological adaptations in pregnancy can serve as theoretical models for furthering our understanding of risk factors for perinatal mental disorders in vulnerable individuals. Recent findings have confirmed that neurobiological interactions are complex, and further translational research is needed to determine how these findings can inform clinical care. In particular, RCTs involving women affected by perinatal mental illness are warranted to bridge the gap between pre-clinical and clinical evidence and clinical practice.

In order to explain why some women are susceptible to psychiatric symptoms, and others are not, recent studies have sought out epigenetic mechanisms such as genetic variations and polymorphisms in the sensitivity of estrogen-receptors and serotonin transporter genes [163]. These genetic variants can be activated by psychosocial stress factors such as migration or single parenthood. However, most of the studies on gene variants have utilized small sample sizes, so further studies are required. Other research on ovarian hormone sensitivity has sought to form theoretical models or create experimental conditions [34,46,47,118] defining varying degrees of sensitivity to ovarian hormonal fluctuations as a biological “trait vulnerability” and attributing specific affective and cognitive reactions to certain menstrual phases. These findings could explain why a history of postpartum depression is a primary risk factor for recurrence in future pregnancies. An improved mechanistic understanding of individual risk profiles could facilitate more tailored prevention and treatment strategies in perinatal psychiatry. Present knowledge could be used to devise risk-stratification models including psychiatric history, prior hormone-sensitive mood episodes, bipolar-spectrum symptoms, sleep sensitivity, obstetric complications, thyroid/autoimmune history, trauma history, breastfeeding goals, and social support.

Based on the present literature review, several recommendations can currently be formulated for clinical practice. Blood work is important for differential diagnosis and detecting underlying somatic health conditions that may present with psychiatric symptoms in the perinatal period, such as thyroid disease or anemia [12]. In the absence of established biomarkers, screening through clinical assessments is essential. ACOG recommends screening all pregnant patients for the most common perinatal conditions, depression and anxiety [12]. Our review supports this recommendation and deems screening particularly important in vulnerable patient populations such as women with inflammatory conditions, unplanned cesarian sections, a history of mental illness, previous adverse mood reactions to contraceptives and other psychosocial risk factors. All health care professionals involved in the care of women during the perinatal period should be trained to recognize signs of suicidality and psychosis since these represent perinatal psychiatric emergencies requiring urgent attention. In psychiatric practice, clinical interviews can help identify women who are particularly susceptible to hormonal instability; counsel them about risks of recurrence during vulnerable time windows; and guide them towards targeted treatments like SSRIs for mood stabilization in premenstrual dysphoric disorder [164] or the new “GABA-kines” for postpartum depression. When prescribing medication to pregnant or lactating women, it is particularly important to discuss potential benefits and risks in a shared decision-making process [14]. Non-pharmacological interventions are also important for the treatment of perinatal illness. Psychotherapeutic treatment is particularly relevant for the treatment of non-psychotic perinatal conditions such as depression, anxiety, PTSD and OCD. Meanwhile, sleep protection is essential for the treatment and prevention of perinatal psychotic disorders. It appears that certain birth and postnatal experiences, including breastfeeding and skin-to-skin contact, could offer some protection against PTSD-FC, postpartum depression, and anxiety when these experiences are desired and positively experienced by the mother. Breastfeeding, rooming-in and access to lactation counseling should be encouraged in hospital practices, while feelings of shame or guilt associated with the personal or medically informed choice not to do so should be addressed with sensitivity. It is important to note that perinatal psychiatry is best provided through interdisciplinary teams where psychiatrists work alongside nurses and psychologists and are in close coordination with obstetric and pediatric specialists, ideally in the setting of psychiatric mother–baby units.

Certain limitations apply to the findings presented. Numerous biological brain studies have merely been conducted on rodents, and it remains questionable whether findings can simply be translated to women. Some of the statements on the evolutionary purpose of biological mechanisms are speculative and have not been scientifically substantiated. Furthermore, the cytokine system in its complexity and the interaction with the placenta and the maternal brain have not been sufficiently explored and should become the subject of future studies [165].

Though research efforts have sought to identify a molecular biomarker as a predictor for postpartum depression, so far, no single predictor has become a routinely employed clinical tool. Lastly, the absence of a systematic review or meta-analysis on this topic should be acknowledged as a limitation. This reflects the broad scope of the review, which synthesizes evidence across multiple physiological mechanisms and subtopics rather than addressing a single, narrowly defined research question. 

Even though rates of birth are on a continuous worldwide decline, perinatal mental illness continues to be a debilitating condition and an urgent international public health issue. Suicide is a leading cause of maternal death in the perinatal period in several high-income settings and is an important preventable contributor to maternal mortality [166].

Further resources into treatment, prevention and studies into its causes are called for and should be supported. Physiological adaptations in pregnancy and postpartum should not be pathologized but instead recognized as potential stress tests for vulnerable neurobiological systems. Clinical care should therefore combine biological assessment, attentive clinical interviews with risk stratification, sleep protection, trauma-informed obstetric practice, breastfeeding support without coercion, and coordinated multidisciplinary follow-up.

## Data Availability

No new data were created or analyzed in this study.
